# Three-year course of clinical high-risk symptoms for psychosis in the community: a latent class analysis

**DOI:** 10.1017/S2045796024000891

**Published:** 2025-01-13

**Authors:** C. Michel, N. Osman, G. Rinaldi, B. G. Schimmelmann, J. Kindler, F. Schultze-Lutter

**Affiliations:** 1 University Hospital of Child and Adolescent Psychiatry and Psychotherapy, University of Bern, Bern, Switzerland; 2 Department of Psychiatry and Psychotherapy, Medical Faculty, Heinrich-Heine-University Düsseldorf, Düsseldorf, Germany; 3 University Hospital of Child and Adolescent Psychiatry, University Hospital Hamburg-Eppendorf, Hamburg, Germany; 4 Child and Adolescent Psychiatry, Psychiatry Baselland, Liestal, Switzerland; 5 Department of Psychology, Faculty of Psychology, Airlangga University, Surabaya, Indonesia

**Keywords:** clinical profiles, community, course, general population, latent class analysis, movement, outcome, psychosis risk

## Abstract

**Aims:**

Clinical high-risk for psychosis (CHR-P) states exhibit diverse clinical presentations, prompting a shift towards broader outcome assessments beyond psychosis manifestation. To elucidate more uniform clinical profiles and their trajectories, we investigated CHR-P profiles in a community sample.

**Methods:**

Participants (*N* = 829; baseline age: 16–40 years) comprised individuals from a Swiss community sample who were followed up over roughly 3 years. latent class analysis was applied to CHR-P symptom data at baseline and follow-up, and classes were examined for demographic and clinical differences, as well as stability over time.

**Results:**

Similar three-class solutions were yielded for both time points. Class 1 was mainly characterized by subtle, subjectively experienced disturbances in mental processes, including thinking, speech and perception (basic symptoms [BSs]). Class 2 was characterized by subthreshold positive psychotic symptoms (i.e., mild delusions or hallucinations) indicative of an ultra-high risk for psychosis. Class 3, the largest group (comprising over 90% of participants), exhibited the lowest probability of experiencing any psychosis-related symptoms (CHR-P symptoms). Classes 1 and 2 included more participants with functional impairment and psychiatric morbidity. Class 3 participants had a low probability of having functional deficits or mental disorders at both time points, suggesting that Class 3 was the healthiest group and that their mental health and functioning remained stable throughout the study period. While 91% of Baseline Class 3 participants remained in their class over time, most Baseline Classes 1 (74%) and Class 2 (88%) participants moved to Follow-up Class 3.

**Conclusions:**

Despite some temporal fluctuations, CHR-P symptoms within community samples cluster into distinct subgroups, reflecting varying levels of symptom severity and risk profiles. This clustering highlights the largely distinct nature of BSs and attenuated positive symptoms within the community. The association of Classes 1 and 2 with Axis-I disorders and functional deficits emphasizes the clinical significance of CHR-P symptoms. These findings highlight the need for personalized preventive measures targeting specific risk profiles in community-based populations.

## Introduction

Early detection and treatment of clinical high-risk for psychosis (CHR-P) states are not only relevant for preventing the onset of the first episode of psychosis, but also for achieving remission of CHR-P symptoms and other comorbidities, and for avoiding impairments in psychosocial functioning (Addington *et al.*, 2019; Caballero *et al.*, 2023; Campion *et al.*, 2012; Schmidt *et al.*, 2015; Schultze-Lutter and Meisenzahl, 2023; Worthington and Cannon, 2021). In clinical samples, many CHR-P patients who do not develop psychosis within follow-up – so-called ‘non-converters’ – do not experience remission from CHR-P symptoms. Furthermore, they continue to suffer from non-psychotic mental disorders at follow-up – mainly mood and anxiety disorders – (Beck *et al.*, 2019), which are the most frequent comorbid disorders reported for CHR-P states at baseline (Solmi *et al.*, 2023). Irrespective of comorbidities, half of clinical CHR-P samples show a poor psychosocial outcome (Carrión *et al.*, 2013; Lin *et al.*, 2015), even when CHR-P symptoms remit (Addington *et al.*, 2019), with CHR-P state at follow-up (either newly developed or maintained) being associated with significantly lower functioning (Lin *et al.*, 2015; Michel *et al.*, 2018a; Schmidt *et al.*, 2015). Therefore, regardless of conversion, the CHR-P state itself clearly possesses clinical significance warranting support and care in help-seeking individuals (Fusar-Poli *et al.*, 2020; Ruhrmann *et al.*, 2010; Solmi *et al.*, 2023).

A challenge to the understanding of CHR-P states and their course is the heterogeneous clinical picture. This difficulty has been tackled by various methods, from identifying specific risk profiles linked to neural mechanisms, to building multivariate models that predict heterogeneous outcomes (Caballero *et al.*, 2023; Solmi *et al.*, 2023; Worthington and Cannon, 2021). A common method to parse out heterogeneity by way of clinical profiles is latent class analysis (LCA) (Healey *et al.*, 2018; Ryan *et al.*, 2018; Valmaggia *et al.*, 2013; van Tricht *et al.*, 2015). Considered a ‘person-centred’ approach to reduce heterogeneity, LCA operates on the notion of finding ‘hidden’ homogenous groups within heterogeneous populations (Rosato and Baer, 2012). Studies applying LCA to clinical CHR-P samples generally used baseline data only, and characterized groups by transition rates to psychosis, while other relevant outcomes (e.g., non-psychotic mental disorders) were not considered (Healey *et al.*, 2018; Ryan *et al.*, 2018; Valmaggia *et al.*, 2013; van Tricht *et al.*, 2015). They have reported between two and five classes differing in parameters included for class selection (e.g., only positive symptoms or additional negative symptoms, or neurophysiological parameters) (Healey *et al.*, 2018; Ryan *et al.*, 2018; Valmaggia *et al.*, 2013; van Tricht *et al.*, 2015). To date, no study has attempted to determine if and how people might change class membership between baseline and follow-up, or examined stability of classes over time. Moreover, earlier studies were carried out in selected samples of only, or mostly, help-seeking CHR-P patients defined exclusively by ultra-high risk (UHR) criteria, who commonly receive treatment (Healey *et al.*, 2018; Ryan *et al.*, 2018; Valmaggia *et al.*, 2013; van Tricht *et al.*, 2015) and who must therefore be assumed a non-representative minority of the CHR-P population. Consequently, the classes and natural course (i.e., potentially without treatment) of clinician-assessed CHR-P symptoms in the wider community using the whole spectrum of CHR-P criteria and symptoms, i.e., both UHR and basic symptom (BS) criteria (Schultze-Lutter *et al.*, 2015), is largely unknown.

To address this gap in knowledge (van Os *et al.*, 2021), the aims of this study were twofold. First, to ascertain CHR-P symptom-based classes of community participants using the whole spectrum of CHR-P symptoms (i.e., attenuated (APS) and brief intermittent psychotic symptoms (BIPS) and criteria-relevant BS), and to examine their clinical and socio-demographic correlates. Second, to explore the stability of these classes longitudinally; specifically, to determine how class membership itself might change, or how individuals might ‘move between’ baseline and follow-up classes.

## Methods

### Participants

**Figure 1.**
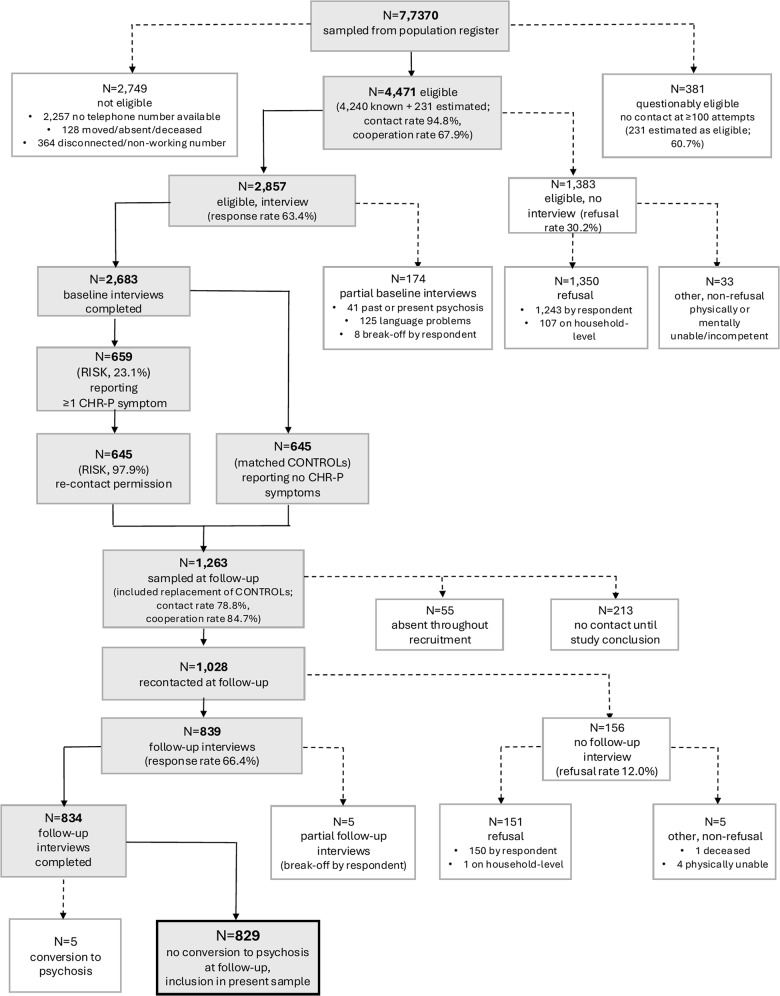
Flowchart of participant recruitment, selection and follow-up in the BEAR study.

The sample included participants from both the baseline and follow-up assessments of the ‘Bern Epidemiological At-Risk’ (BEAR) study (Schultze-Lutter *et al.*, 2018, 2021; for further details, see eTexts 1, 2). At baseline, we evaluated CHR-P symptoms and criteria in a representative random sample of the 16- to 40-year-old Bernese community (*N* = 2,683; response rate: 63.4%), using procedures comparable with clinical assessment (Schultze-Lutter *et al.*, 2015). A selected, CHR-P symptom-enriched sample (*N* = 834; response rate: 66.4%) was followed up approximately 3 years later, and only the *N* = 829 non-converters were included in the present analyses (Schultze-Lutter *et al.*, 2021). For a detailed overview of the participant selection process, including reasons for exclusion, please refer to Fig. 1. Participation was voluntary and required informed consent at each time point. The human research ethics committee of Canton Bern approved the study (ID PB_2018-00132).

### Assessments

CHR-P symptoms (eTable 1) were assessed using semi-structured interviews with good interrater reliability (McGlashan *et al.*, 2010; Schultze-Lutter *et al.*, 2007). The Structured Interview for Psychosis-Risk Syndromes (SIPS) (McGlashan *et al.*, 2010) was used for UHR symptoms, i.e., five APS/BIPS, and the Schizophrenia Proneness Instrument, Adult version (Schultze-Lutter *et al.*, 2007) for the 14 BS included in the two BS criteria (Schultze-Lutter *et al.*, 2015). For the present analyses, CHR-P symptoms were defined by the presence of APS or BIPS, and/or criteria-relevant BS at baseline, irrespective of the onset/worsening and/or frequency requirements of related CHR-P criteria. The five positive SIPS-items were recoded into binary items: 1 (presence) was assigned to scores between 3 and 6 (indicating presence of APS or BIPS) and 0 (absence) to scores between 0 and 2 (indicating absence of APS and BIPS). Similarly, BS-scores between 1 and 6, and 8 (indicating presence of BS) were recoded as 1 (presence), while 0 (absence) was assigned to BS-scores of 0, 9 and 7 (respectively indicating absence of BS, their only questionable presence, or that the symptom has always been present in the same frequency, making it a trait feature, not a BS). Present DSM-IV non-substance-related axis-I disorders, including affective, anxiety (including specific phobia), eating, somatoform, obsessive-compulsive and post-traumatic stress disorder were assessed using the Mini-International Neuropsychiatric Interview (Sheehan *et al.*, 1998), which was previously successfully applied in telephone interviews of community samples, demonstrating good reliability, concurrent and predictive validity (Schultze-Lutter *et al.*, 2018; Sheehan *et al.*, 1998; Wang *et al.*, 2006).

Clinician-rated global level of psychosocial functioning, independent of overall symptom severity, was estimated on the Social and Occupational Functioning Assessment Scale (SOFAS; American Psychiatric Association (APA), 1994), which showed good psychometric properties, including good interrater reliability and construct validity (Hilsenroth *et al.*, 2000; Rybarczyk, 2011). Over a 0–100 range, lower SOFAS-scores represent lower functioning, with a score of ≤70 indicating presence of a functional deficit (American Psychiatric Association (APA), 1994; Morosini *et al.*, 2000; Schimmelmann *et al.*, 2015; Michel *et al.*, 2018b).

### Statistical analyses

Analyses were conducted in R (Version 4.2) and RStudio (Version 2022.07.0). To identify the best fitting LCA model for each assessment point, different models were estimated, and subsequent classes were added using the R package poLCA (Linzer and Lewis, 2011). For each model, the Akaike Information Criterion (AIC), the Bayesian Information Criterion (BIC) and the relative entropy were calculated. Lower AIC and BIC values indicate better fit, and higher entropy values indicate better accuracy with the defined classes (Weller *et al.*, 2020). After identifying the best-fitting LCA model for both baseline and follow-up data, each individual was assigned to a specific class based on the probabilities of class membership obtained from the analysis.

Differences between classes regarding ratio data and categorical variables were tested using ANOVAs and chi-squared tests, respectively. Effect sizes were calculated using eta-square and Cramer’s *V*. Significant ANOVAs were additionally tested using pairwise Bonferroni corrected comparisons. For significant chi-squared tests, the standardized residuals (≥|1.96|) were calculated as a measure of significant cell difference between observed and expected values.

## Results

### Sample characteristics at baseline and follow-up

The mean follow-up time was 40.60 months (SD = 8.35, Mdn = 39.00, range: 21.00–68.00). Participants were on average 29.8 years old at baseline and 33.3 years old at follow-up (eTable 2). At both time points, the sample was 53.2% female, predominantly Swiss and in regular employment (>95%), with most participants (84.1%) pursuing or holding moderate to high educational qualifications (ISCED ≥ 4); roughly half of the sample was single (eTable 2). At both time points, the proportion of participants with a functional deficit remained stable at around 7%, while the rate of axis-I disorders significantly decreased from 17.0% at baseline to 13.3% at follow-up, primarily due to reductions in affective and other disorders (e.g., eating disorders, somatoform disorders; eTable 2). All symptoms decreased in number over time or maintained a low frequency (eTable 2), except for perceptual symptoms, which showed an increase at follow-up (eTable 2).

### LCA at baseline

At baseline, three LCA models were tested and compared by goodness of fit.

Although a two-class solution showed the best BIC, which is generally considered the most reliable fit statistic in LCA (Sinha *et al.*, 2021; Weller *et al.*, 2020), its AIC and entropy value were the poorest. Therefore, the two-class solution was discarded.

**Figure 2.**
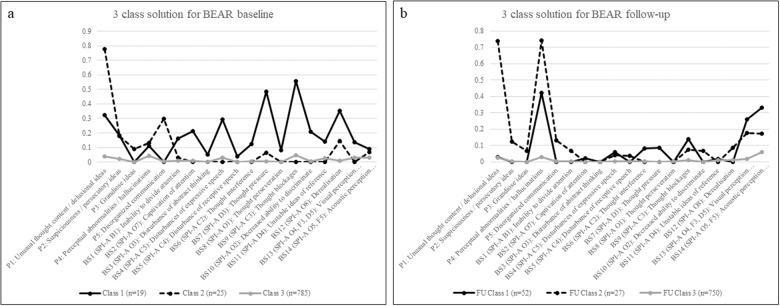
Latent class profiles of basic symptoms and (attenuated) psychotic symptoms at baseline (a) and follow-up (b).

Overall, the best fitting model was a three-class solution (eTable 3), showing the lowest AIC, the second-lowest BIC and the second-highest entropy (Fig. 2a), indicating clear separation between the classes. Classes 1 and 2 of the three-class solution were mostly characterized by a high probability of BS and of APS/BIPS, respectively. Class 3 was characterized by a low probability of any CHR-P symptom (Fig. 2a).

### Baseline characteristics of the Baseline Classes

Baseline Class 1 was the smallest (*n* = 19, 2.3%), including participants who, compared to the other classes, showed the highest rate of lower education level as well as unemployment or sheltered/temporary employment. Additionally, they showed high rates of functional impairment and were the most affected by axis-I disorders (Table 1). In comparison, Baseline Class 2 was slightly bigger (*n* = 25, 3.0%), comprising individuals with similar rates of functional impairment, but fewer, although still frequent, axis-I disorders (Table 1). Baseline Class 2 members were also the oldest, and least likely to be single. Finally, Baseline Class 3 was the largest (*n* = 785, 94.7%), characterized by the highest rate of regular full-time/part-time employment, and the lowest rates of psychosocial deficits, axis-I disorders and divorce (Table 1).

**Table 1.** Baseline socio-demographic and clinical characteristics of the three Baseline Classes (*N* = 829)

		Baseline Class 1 (*n* = 19)		Baseline Class 2 (*n* = 25)		Baseline Class 3 (*n* = 785)		
		*n*	% [significant standardized residuals]	*n*	% [significant standardized residuals]	*n*	% [significant standardized residuals]	Statistics
Age (mean ± SD, median, range)		27.53 ± 8.82, 23, 18−39		**35.08 ± 5.09, 37, 19−40**		29.71 ± 7.66, 32. 15−41		*F* = 6.877, df = 2, ***p* = 0.0011**, *η*^2^ = 0.016
								*Bonferroni adjusted:*
								***Class 1 vs. Class 2:* p* = 0.004, Class 2 vs. Class 3:* p* = 0.002***
								*Class 1 vs. Class 3:* p* = 0.651*
Sex (male)		6	31.6	15	60.0	367	46.8	*χ*^2^ = 3.5183, df = 2, *p* = 0.172, Cramer’s *V* = 0.065
Nationality (Swiss)		19	100.0	24	96.0	755	96.2	*χ*^2^ = 0.75755, df = 2, *p* = 0.685, Cramer’s *V* = 0.030
Highest education								
	ISCED level 0–2	1	5.3	0	0.0	14	1.8	*χ*^2^ = 11.153, df = 8, *p* = 0.193, Cramer’s *V* = 0.082
	ISCED level 3	**6**	**31.6 [2.21]**	2	8.0	109	13.9	
	ISCED level 4–5	9	47.4	16	64.0	367	46.8	
	ISCED level 7	3	15.8	7	28.0	287	36.6	
	ISCED level 8	0	0.0	0	0.0	8	1.0	
Current employment								
	Unemployed	**3**	**15.8 [3.72]**	1	4.0	**17**	**2.2 [−2.84]**	*χ*^2^ = 119.86, df = 8, ***p* < 0.001**, Cramer’s *V* = 0.269
	Sheltered employment	**1**	**5.3 [6.53]**	0	0.0	**0**	**0.0 [−4.23]**	
	Temporary employment	**2**	**10.5 [4.31]**	0	0.0	**6**	**0.8 [−2.50]**	
	Regular full- and part-time employment	**12**	**63.2 [−7.69]**	24	96.0	**762**	**97.1 [5.19]**	
	Other	**1**	**5.3 [6.53]**	0	0.0	**0**	**0.0 [−4.23]**	
Marital status								
	Single	12	63.2	**9**	**36.0 [−2.12]**	449	57.2	*χ*^2^ = 14.776, df = 10, *p* = 0.141, Cramer’s *V* = 0.094
	Married/civil union	7	36.8	13	52.0	315	40.1	
	Separated	0	0.0	1	4.0	10	1.3	
	Divorced	0	0.0	**2**	**8.0 [3.16]**	**8**	**1.0 [−2.09]**	
	Widowed	0	0.0	0	0.0	1	0.1	
	Other	0	0.0	0	0.0	2	0.3	
Family history of psychiatric disorders		11	57.9	12	48.0	329	42.1	*χ*^2^ = 2.205, df = 2, *p* = 0.332 Cramer’s *V* = 0.052
SOFAS deficit (SOFAS < 70)		**9**	**47.4 [6.69]**	**12**	**48.0 [7.82]**	**41**	**7.5 [−10.43]**	*χ*^2^ = 108.79, df = 2, ***p* < 0.001**, Cramer’s *V* = 0.362
Any current axis-I disorder		**11**	**57.9 [4.80]**	**10**	**40.0 [3.11]**	**120**	**15.3 [−5.57]**	*χ*^2^ = 33.512, df = 2, ***p* < 0.001**, Cramer’s *V* = 0.201
	Any affective disorder	**9**	**47.4 [7.30]**	**7**	**28.0 [4.42]**	**38**	**4.8 [−8.25]**	*χ*^2^ = 74.638, df = 2, ***p* < 0.001**, Cramer’s *V* = 0.300
	Any anxiety disorder (including specific phobia)	**8**	**42.1 [4.17]**	**8**	**32.0 [3.21]**	**81**	**10.3 [−5.23]**	*χ*^2^ = 28.423, df = 2, ***p* < 0.001**, Cramer’s *V* = 0.185
	Other disorder	**6**	**31.6 [5.89]**	**5**	**20.0 [3.90]**	**25**	**3.2 [−6.91]**	*χ*^2^ = 51.218, df = 2, ***p* < 0.001**, Cramer’s *V* = 0.249
CHR-P symptoms								
	P1: Unusual thought content/delusional ideas	**7**	**36.8 [4.65]**	**23**	**92.0 [15.63]**	**37**	**4.7 [−15.03]**	*χ*^2^ = 270.14, df = 2, ***p* < 0.001**, Cramer’s *V* = 0.571
	P2:Suspiciousness/persecutory ideas	**4**	**21.1 [4.21]**	**7**	**28.0 [6.77]**	**18**	**2.3 [−7.98]**	*χ*^2^ = 65.18, df = 2, ***p* < 0.001**, Cramer’s *V* = 0.280
	P3: Grandiose ideas	0	0.0	**3**	**12.0 [8.44]**	**1**	**0.1 [−6.23]**	*χ*^2^ = 71.22, df = 2, ***p* < 0.001**, Cramer’s *V* = 0.293
	P4: Perceptual abnormalities/hallucinations	2	10.5	**6**	**24.0 [4.38]**	**34**	**4.3 [−4.08]**	*χ*^2^ = 20.693, df = 2, ***p* < 0.001**, Cramer’s *V* = 0.158
	P5: Disorganized communication	0	0.0	**10**	**40.0 [14.55]**	**5**	**0.6 [−10.70]**	*χ*^2^ = 211.66, df = 2, ***p* < 0.001**, Cramer’s *V* = 0.505
	BS1: Inability to divide attention (SPI-A B1)	**4**	**21.1 [10.58]**	**1**	**4.0 [1.96]**	**1**	**0.1 [−8.56]**	*χ*^2^ = 116.90, df = 2, ***p* < 0.001**, Cramer’s *V* = 0.376
	BS2: Captivation of attention by details of the visual field (SPI-A O7)	**5**	**26.3 [9.63]**	0	0.0	**6**	**0.8 [−5.98]**	*χ*^2^ = 92.851, df = 2, ***p* < 0.001**, Cramer’s *V* = 0.335
	BS3: Disturbances of abstract thinking (SPI-A O3)	**1**	**5.3 [3.60]**	0	0.0	**2**	**0.3 [−2.17]**	*χ*^2^ = 12.999, df = 2, ***p* < 0.001**, Cramer’s *V* = 0.125
	BS4: Disturbances of expressive speech (SPI-A C5)	**6**	**31.6 [6.35]**	0	0.0	**26**	**3.3 [−3.46]**	*χ*^2^ = 40.976, df = 2, ***p* < 0.001**, Cramer’s *V* = 0.222
	BS5: Disturbances of receptive speech (SPI-A C4)	**1**	**5.3 [6.53]**	0	0.0	**0**	**0.0 [−4.23]**	*χ*^2^ = 42.683, df = 2, ***p* < 0.001**, Cramer’s *V* = 0.227
	BS6: Thought interference (SPI-A C2)	**3**	**15.8 [6.69]**	0	0.0	**5**	**0.6 [−4.08]**	*χ*^2^ = 44.818, df = 2, ***p* < 0.001**, Cramer’s *V* = 0.232
	BS7: Thought pressure (SPI-A D3)	**11**	**57.9 [16.86]**	**3**	**12.0 [3.42]**	**4**	**0.5 [−13.87]**	*χ*^2^ = 299.32, df = 2, ***p* < 0.001**, Cramer’s *V* = 0.601
	BS8: Thought perseveration (SPI-A O1)	**2**	**10.5 [9.24]**	0	0.0	**0**	**0.0 [−5.98]**	*χ*^2^ = 85.469, df = 2, ***p* < 0.001**, Cramer’s *V* = 0.321
	BS9: Thought blockages (SPI-A C3)	**11**	**57.9 [9.72]**	0	0.0	**38**	**4.8 [−5.52]**	*χ*^2^ = 95.51, df = 2, ***p* < 0.001**, Cramer’s *V* = 0.339
	BS10: Decreased ability to discriminate between ideas & perception, fantasy & true memories (SPI-A O2)	**5**	**26.3 [10.74]**	0	0.0	**4**	**0.5 [−6.76]**	*χ*^2^ = 115.33, df = 2, ***p* < 0.001**, Cramer’s *V* = 0.373
	BS11: Unstable ideas of reference (SPI-A D4)	**3**	**15.8 [3.29]**	0	0.0	22	2.8	*χ*^2^ = 11.499, df = 2, ***p* < 0.001**, Cramer’s *V* = 0.118
	BS12: Derealization (SPI-A O8)	**9**	**47.4 [12.92]**	**5**	**20.0 [5.82]**	**6**	**0.8 [−13.06]**	*χ*^2^ = 205.00, df = 2, ***p* < 0.001**, Cramer’s *V* = 0.497
	BS13: Visual perception disturbances (SPI-A O4, F3, D5)	**3**	**15.8 [3.03]**	0	0.0	25	3.2	*χ*^2^ = 9.933, df = 2, ***p* < 0.001**, Cramer’s *V* = 0.110
	BS14: Acoustic perception disturbances (SPI-A O5, F5)	2	10.5	2	8.0	25	3.2	*χ*^2^ = 4.509, df = 2, *p* = 0.105, Cramer’s *V* = 0.074

*Note:* SOFAS: Social and Occupational Functioning Assessment Scale. In **[bold]**, cells with standardized residuals ≥|1.96|. This equals significant deviation from the expected cell frequency. An adjusted residual of 1.96 indicates that the number of cases in that cell is significantly larger than would be expected if the null hypothesis were true, with a significance level of 0.05. An adjusted residual that is <−1.96 indicates that the number of cases in that cell is significantly smaller than would be expected if the null hypothesis were true. P: positive-symptom scale; BS: basic symptom.

There were no differences between the Baseline Classes in terms of sex, nationality or family history of mental disorders.

### Follow-up characteristics of the Baseline Classes

At follow-up, participants in Baseline Class 1 continued to show the highest rates of unemployment or sheltered/temporary employment, lower education level and axis-I disorders. Newly, they showed the highest rates of functional impairment (Table 2). Baseline Class 2 remained the oldest, showing intermediate rates of functional deficits and any axis-I disorder. Among its members, rates of regular full- or part-time employment decreased compared to baseline, while other types of employment were now highly frequent (Table 2). Finally, participants in Baseline Class 3 continued to report the highest levels of education and regular full- or part-time employment, as well as the lowest rates of axis-I disorders and functional impairment (Table 2).

**Table 2.** Follow-up socio-demographic and clinical characteristics of the three Baseline Classes (*N* = 829)

		Baseline Class 1 (*n* = 19)		Baseline Class 2 (*n* = 25)		Baseline Class 3 (*n* = 785)		
		*n*	% [significant standardized residuals]	*n*	% [significant standardized residuals]	*n*	% [significant standardized residuals]	Statistics
Age (mean ± SD, median, range)		30.95 ± 9.1, 26, 21–45		**38.64 ± 5.15, 39, 22–45**		33.14 ± 7.75, 35, 19–45		*F* = 7.029, df = 2, ***p* < 0.001**, *η*^2^ = 0.017
								*Bonferroni adjusted:*
								***Class 1 vs. Class 2:* p* = 0.003, Class 2 vs. Class 3:* p* = 0.001***
								*Class 1 vs. Class 3:* p* = 0.668*
Sex (male)		6	31.6	15	60.0	367	46.8	*χ*^2^ = 3.5183, df = 2, *p* = 0.172, Cramer’s *V* = 0.065
Nationality (Swiss)		19	100.0	24	96.0	762	97.1	*χ*^2^ = 0.678, df = 2, *p* = 0.712, Cramer’s *V* = 0.029
Highest education								
	ISCED level 0–2	1	5.3	0	0.0	14	1.8	*χ*^2^ = 11.153, df = 8, *p* = 0.193, Cramer’s *V* = 0.082
	ISCED level 3	**6**	**31.6 [2.21]**	2	8.0	109	13.9	
	ISCED level 4–5	9	47.4	16	64.0	367	46.8	
	ISCED level 7	3	15.8	7	28.0	287	36.6	
	ISCED level 8	0	0.0	0	0.0	8	1.0	
Current employment								
	Unemployed	**3**	**15.8 [3.98]**	1	4.0	**15**	**1.9 [−3.10]**	*χ*^2^ = 67.632, df = 8, ***p* < 0.001**, Cramer’s *V* = 0.202
	Sheltered employment	**2**	**10.5 [6.39]**	0	0.0	**2**	**0.3 [−4.00]**	
	Temporary employment	0	0.0	1	4.0	6	0.8	
	Regular full- and part-time employment	**14**	**73.7 [−4.94]**	**22**	**88.0 [−2.02]**	**759**	**96.7 [4.84]**	
	Other	0	0.0	**1**	**4.0 [2.58]**	3	0.4	
Marital status								
	Single	12	63.2	8	32.0	404	51.5	*χ*^2^ = 13.519, df = 10, *p* = 0.109, Cramer’s *V* = 0.090
	Married/civil union	7	36.8	13	52.0	348	44.3	
	Separated	0	0.0	**2**	**8.0 [2.49]**	12	1.5	
	Divorced	0	0.0	2	8.0	17	2.2	
	Widowed	0	0.0	0	0.0	2	0.3	
	Other	0	0.0	0	0.0	2	0.3	
Family history of psychiatric disorders		12	63.2	12	48.0	388	49.4	*χ*^2^ = 1.637, df = 2, *p* = 0.802, Cramer’s *V* = 0.031
SOFAS deficit (SOFAS < 70)		**7**	**36.8 [5.16]**	**5**	**20.0 [2.59]**	**46**	**5.9 [−5.42]**	*χ*^2^ = 34.065, df = 2, ***p* < 0.001**, Cramer’s *V* = 0.203
Any current axis-I disorder		**9**	**47.4 [4.43]**	5	20.0	**96**	**12.2 [−3.73]**	*χ*^2^ = 33.512, df = 2, ***p* < 0.001**, Cramer’s *V* = 0.201
	Any affective disorder	**6**	**31.6 [8.38]**	**4**	**16.0 [4.50]**	**10**	**1.3 [−9.02]**	*χ*^2^ = 92.579, df = 2, ***p* < 0.001**, Cramer’s *V* = 0.334
	Any anxiety disorder (including specific phobia)	**6**	**31.6 [2.70]**	3	12.0	89	11.3	*χ*^2^ = 7.292, df = 2, ***p* = 0.026**, Cramer’s *V* = 0.094
	Any other disorder	**2**	**10.5 [3.18]**	**2**	**8.0 [2.63]**	**9**	**1.1 [−4.13]**	*χ*^2^ = 17.482, df = 2, ***p* < 0.001**, Cramer’s *V* = 0.145
CHR-P symptoms								
	P1: Unusual thought content/delusional ideas	2	10.5	**5**	**20.0 [3.33]**	**37**	**4.7 [−3.22]**	*χ*^2^ = 17.482, df = 2, ***p* = 0.002**, Cramer’s *V* = 0.122
	P2:Suspiciousness/persecutory ideas	0	0.0	0	0.0	6	0.8	*χ*^2^ = 0.339, df = 2, *p* = 0.844, Cramer’s *V* = 0.020
	P3: Grandiose ideas	0	0.0	**2**	**8.0 [8.03]**	**0**	**0.0 [−5.98]**	*χ*^2^ = 64.476, df = 2, ***p* < 0.001**, Cramer’s *V* = 0.279
	P4: Perceptual abnormalities/hallucinations	**5**	**26.3 [2.31]**	**6**	**24.0 [2.27]**	**75**	**9.6 [−3.27]**	*χ*^2^ = 10.753, df = 2, ***p* = 0.005**, Cramer’s *V* = 0.114
	P5: Disorganized communication	0	0.0	**3**	**12.0 [7.47]**	**2**	**0.3 [−5.47]**	*χ*^2^ = 55.87, df = 2, ***p* < 0.001**, Cramer’s *V* = 0.260
	BS1: Inability to divide attention (SPI-A B1)	0	0.0	0	0.0	4	0.5	*χ*^2^ = 0.225, df = 2, *p* = 0.894, Cramer’s *V* = 0.016
	BS2: Captivation of attention by details of the visual field (SPI-A O7)	0	0.0	0	0.0	2	0.3	*χ*^2^ = 0.112, df = 2, *p* = 0.945, Cramer’s *V* = 0.012
	BS3: Disturbances of abstract thinking (SPI-A O3)	0	0.0	0	0.0	0	0.0	—
	BS4: Disturbances of expressive speech (SPI-A C5)	1	5.3	0	0.0	8	1.0	*χ*^2^ = 3.395, df = 2, *p* = 0.183, Cramer’s *V* = 0.064
	BS5: Disturbances of receptive speech (SPI-A C4)	0	0.0	**1**	**4.0 [2.58]**	3	0.4	*χ*^2^ = 6.698, df = 2, ***p* = 0.035**, Cramer’s *V* = 0.090
	BS6: Thought interference (SPI-A C2)	1	5.3	0	0.0	8	1.0	*χ*^2^ = 3.395, df = 2, *p* = 0.183, Cramer’s *V* = 0.064
	BS7: Thought pressure (SPI-A D3)	0	0.0	1	4.0	8	1.0	*χ*^2^ = 2.218, df = 2, *p* = 0.330 Cramer’s *V* = 0.052
	BS8: Thought perseveration (SPI-A O1)	0	0.0	0	0.0	1	0.1	*χ*^2^ = 0.056, df = 2, *p* = 0.972, Cramer’s *V* = 0.008
	BS9: Thought blockages (SPI-A C3)	**3**	**15.8 [3.39]**	1	4.0	**20**	**2.5 [−2.52]**	*χ*^2^ = 11.683, df = 2, ***p* = 0.003**, Cramer’s *V* = 0.119
	BS10: Decreased ability to discriminate between ideas & perception, fantasy & true memories (SPI-A O2)	0	0.0	0	0.0	2	0.3	*χ*^2^ = 0.112, df = 2, *p* = 0.945, Cramer’s *V* = 0.012
	BS11: Unstable ideas of reference (SPI-A D4)	0	0.0	0	0.0	9	1.1	*χ*^2^ = 0.51, df = 2, *p* = 0.775, Cramer’s *V* = 0.025
	BS12: Derealization (SPI-A O8)	**3**	**15.8 [6.26]**	1	4.0	**5**	**0.6 [−5.27]**	*χ*^2^ = 41.702, df = 2, ***p* < 0.001**, Cramer’s *V* = 0.224
	BS13: Visual perception disturbances (SPI-A O4, F3, D5)	2	10.5	3	12.0	38	4.5	*χ*^2^ = 3.652, df = 2, *p* = 0.161, Cramer’s *V* = 0.066
	BS14: Acoustic perception disturbances (SPI-A O5, F5)	2	10.5	2	8.0	75	9.6	*χ*^2^ = 0.090, df = 2, *p* = 0.956, Cramer’s *V* = 0.010

*Note:* SOFAS: Social and Occupational Functioning Assessment Scale. In **[bold]**, cells with standardized residuals ≥|1.96|. This equals significant deviation from the expected cell frequency. An adjusted residual of 1.96 indicates that the number of cases in that cell is significantly larger than would be expected if the null hypothesis were true, with a significance level of 0.05. An adjusted residual that is <−1.96 indicates that the number of cases in that cell is significantly smaller than would be expected if the null hypothesis were true. P: positive-symptom scale; BS: basic symptom.

### New LCA for the follow-up time point

For the follow-up data, three new LCA models were tested and compared by goodness of fit.

Again, a three-class solution was the best fitting model, showing the lowest AIC and BIC values, yet had relatively low relative entropy (see eTable 4, Fig. 2b), indicating higher within-classes homogeneity at this time point compared to baseline.

### Follow-up characteristics of Follow-up Classes

Overall, Follow-up Class 1 (6.3% of sample) resembled Baseline Class 1, showing the highest rates of lower education and axis-I disorders. However, Follow-up Class 1 members showed only an intermediate rate of functional deficits and had the highest rate of separated persons (Table 3). With the exception of four BS, they showed a high likelihood of perceptual and cognitive BS, and of perceptual abnormalities/hallucinations (P4).

**Table 3.** Follow-up socio-demographic and clinical characteristics of the three Follow-up Classes (*N* = 829)

		Follow-up Class 1 (*n* = 52)		Follow-up Class 2 (*n* = 27)		Follow-up Class 3 (*n* = 750)		
		*n*	% [significant standardized residuals]	*n*	% [significant standardized residuals]	*n*	% [significant standardized residuals]	Statistics
Age (mean ± SD, median, range)		33.38 ± 7.98, 35, 19–44		32.33 ± 8.28, 33, 19–43		33.27 ± 7.75, 35, 19–44		*F* = 0.199, df = 2, *p* = 0.820, *η*^2^ = 0.000
Sex (male)		18	34.6	12	44.4	358	47.7	*χ*^2^ = 3.423, df = 2, *p* = 0.181, Cramer’s *V* = 0.064
Nationality (Swiss)		52	100.0	26	96.3	727	96.9	*χ*^2^ = 1.692, df = 2, *p* = 0.429, Cramer’s *V* = 0.045
Highest education								
	ISCED level 0–2	**4**	**7.7 [3.29]**	1	3.7	**10**	**1.3 [−3.17]**	*χ*^2^ = 24.191, df = 8, ***p* = 0.002**, Cramer’s *V* = 0.121
	ISCED level 3	7	13.5	3	11.1	107	14.3	
	ISCED level 4–5	30	57.7	**19**	**70.4 [2.44]**	**343**	**45.7 [−2.76]**	
	ISCED level 7	**11**	**21.2 [−2.28]**	**4**	**14.8 [−2.31]**	**282**	**37.6 [3.28]**	
	ISCED level 8	0	0.0	0	0.0	8	1.1	
Current employment								
	Unemployed	2	3.8	2	7.4	15	2.0	*χ*^2^ = 16.527, df = 8, ***p* = 0.035**, Cramer’s *V* = 0.121
	Sheltered employment	0	0.0	0	0.0	4	0.5	
	Temporary employment	0	0.0	1	3.7	6	0.8	
	Regular full- and part-time employment	49	94.2	**23**	**85.2 [−2.85]**	**723**	**95.9 [2.24]**	
	Other	1	1.9	**1**	**3.7 [2.46]**	**2**	**0.5 [−2.76]**	
Marital status								
	Single	24	46.2	18	66.7	382	50.9	*χ*^2^ = 16.408, df = 10, *p* = 0.089, Cramer’s *V* = 0.099
	Married/civil union	22	42.3	**7**	**25.9 [−1.96]**	339	45.2	
	Separated	**3**	**5.8 [2.36]**	0	0.0	11	1.5	
	Divorced	3	5.8	2	7.4	**14**	**1.9 [−2.52]**	
	Widowed	0	0.0	0	0.0	2	0.3	
	Other	0	0.0	0	0.0	2	0.3	
Family history of psychiatric disorders		28	53.8	14	51.9	370	49.3	*χ*^2^ = 0.812, df = 2, *p* = 0.937, Cramer’s *V* = 0.022
SOFAS deficit (SOFAS < 70)		**8**	**15.4 [2.45]**	**8**	**29.6 [4.69]**	**42**	**5.6 [−4.86]**	*χ*^2^ = 29.127, df = 2, ***p* < 0.001**, Cramer’s *V* = 0.187
Any current axis-I disorder		**19**	**36.5 [5.11]**	**8**	**29.6 [2.55]**	**83**	**11.1 [−5.76]**	*χ*^2^ = 33.907, df = 2, ***p* < 0.001**, Cramer’s *V* = 0.202
	Any affective disorder	**4**	**7.7 [2.56]**	**5**	**18.5 [5.55]**	**11**	**1.5 [−5.47]**	*χ*^2^ = 38.756, df = 2, ***p* < 0.001**, Cramer’s *V* = 0.216
	Any anxiety disorder (including specific phobia)	**17**	**32.7 [4.81]**	**8**	**29.6 [2.91]**	**73**	**9.7 [−5.74]**	*χ*^2^ = 33.081, df = 2, ***p* < 0.001**, Cramer’s *V* = 0.200
	Any other disorder	2	3.8	**2**	**7.4 [2.48]**	**9**	**1.2 [−2.63]**	*χ*^2^ = 8.371, df = 2, ***p* = 0.015**, Cramer’s *V* = 0.100
CHR-P symptoms								
	P1: Unusual thought content/delusional ideas	1	1.9	**23**	**85.2 [18.82]**	**20**	**2.7 [−10.45]**	*χ*^2^ = 354.36, df = 2, ***p* < 0.001**, Cramer’s *V* = 0.654
	P2:Suspiciousness/persecutory ideas	0	0.0	**4**	**14.8 [8.78]**	**2**	**0.3 [−4.78]**	*χ*^2^ = 77.172, df = 2, ***p* < 0.001**, Cramer’s *V* = 0.305
	P3: Grandiose ideas	0	0.0	**2**	**7.4 [7.72]**	**0**	**0.0 [−4.36]**	*χ*^2^ = 59.551, df = 2, ***p* < 0.001**, Cramer’s *V* = 0.268
	P4: Perceptual abnormalities/hallucinations	**31**	**59.6 [12.03]**	**22**	**81.5 [12.32]**	**33**	**4.4 [−17.38]**	*χ*^2^ = 311.23, df = 2, ***p* < 0.001**, Cramer’s *V* = 0.613
	P5: Disorganized communication	0	0.0	**4**	**14.8 [9.70]**	**1**	**0.1 [−5.38]**	*χ*^2^ = 94.04, df = 2, ***p* < 0.001**, Cramer’s *V* = 0.337
	BS1: Inability to divide attention (SPI-A B1)	0	0.0	**2**	**7.4 [5.28]**	**2**	**0.3 [−2.76]**	*χ*^2^ = 27.944, df = 2, ***p* < 0.001**, Cramer’s *V* = 0.184
	BS2: Captivation of attention by details of the visual field (SPI-A O7)	**2**	**3.8 [5.47]**	0	0.0	**0**	**0.0 [−4.36]**	*χ*^2^ = 29.957, df = 2, ***p* < 0.001**, Cramer’s *V* = 0.190
	BS3: Disturbances of abstract thinking (SPI-A O3)	0	0.0	0	0.0	0	0.0	—
	BS4: Disturbances of expressive speech (SPI-A C5)	**5**	**9.6 [6.13]**	1	3.7	**3**	**0.4 [−5.87]**	*χ*^2^ = 40.238, df = 2, ***p* < 0.001**, Cramer’s *V* = 0.220
	BS5: Disturbances of receptive speech (SPI-A C4)	0	0.0	**1**	**3.7 [2.46]**	3	0.4	*χ*^2^ = 6.193, df = 2, ***p* = 0.045**, Cramer’s *V* = 0.086
	BS6: Thought interference (SPI-A C2)	**9**	**17.3 [11.66]**	0	0.0	**0**	**0.0 [−9.29]**	*χ*^2^ = 135.96, df = 2, ***p* < 0.001**, Cramer’s *V* = 0.405
	BS7: Thought pressure (SPI-A D3)	**9**	**17.3 [11.66]**	0	0.0	**0**	**0.0 [−9.29]**	*χ*^2^ = 135.96, df = 2, ***p* < 0.001**, Cramer’s *V* = 0.405
	BS8: Thought perseveration (SPI-A O1)	0	0.0	0	0.0	1	0.1	*χ*^2^ = 0.105, df = 2, *p* = 0.949, Cramer’s *V* = 0.011
	BS9: Thought blockages (SPI-A C3)	**12**	**23.1 [8.97]**	2	7.4	**10**	**1.3 [−8.26]**	*χ*^2^ = 83.803, df = 2, ***p* < 0.001**, Cramer’s *V* = 0.318
	BS10: Decreased ability to discriminate between ideas & perception, fantasy & true memories (SPI-A O2)	0	0.0	**2**	**7.4 [7.72]**	**0**	**0.0 [−4.36]**	*χ*^2^ = 59.551, df = 2, ***p* < 0.001**, Cramer’s *V* = 0.268
	BS11: Unstable ideas of reference (SPI-A D4)	2	3.8	0	0.0	7	0.9	*χ*^2^ = 4.148, df = 2, *p* = 0.126, Cramer’s *V* = 0.071
	BS12: Derealization (SPI-A O8)	0	0.0	**3**	**11.1 [5.11]**	**6**	**0.8 [−2.45]**	*χ*^2^ = 26.412, df = 2, ***p* < 0.001**, Cramer’s *V* = 0.179
	BS13: Visual perception disturbances (SPI-A O4, F3, D5)	**21**	**40.4 [11.82]**	**5**	**18.5 [3.18]**	**17**	**2.3 [−11.68]**	*χ*^2^ = 153.76, df = 2, ***p* < 0.001**, Cramer’s *V* = 0.431
	BS14: Acoustic perception disturbances (SPI-A O5, F5)	**26**	**50.0 [10.27]**	5	18.5	**48**	**6.4 [−9.46]**	*χ*^2^ = 109.84, df = 2, ***p* < 0.001**, Cramer’s *V* = 0.364

*Note:* SOFAS: Social and Occupational Functioning Assessment Scale. In **[bold]**, cells with standardized residuals ≥|1.96|. This equals significant deviation from the expected cell frequency. An adjusted residual of 1.96 indicates that the number of cases in that cell is significantly larger than would be expected if the null hypothesis were true, with a significance level of 0.05. An adjusted residual that is <−1.96 indicates that the number of cases in that cell is significantly smaller than would be expected if the null hypothesis were true. P: positive-symptom scale; BS: basic symptom.

Follow-up Class 2 (3.3% of sample) partially resembled Baseline Class 2, showing an intermediate rate of axis-I disorders and the highest probability of all APS/BIPS at follow-up (Table 3). In contrast with Baseline Class 2, members of Follow-up Class 2 additionally had the highest probability of exhibiting four BS (inability to divide attention, disturbance of receptive speech, derealization and decreased ability to discriminate between ideas & perception, fantasy & true memories), as well as an elevated rate of visual perception disturbances, which was, however, still lower than in Follow-up Class 1. Further, they showed the highest rates of psychosocial deficits among Follow-up Classes, as well as the lowest rate of regular full- and part-time employment, and of married persons (Table 3). Overall, Follow-up Class 2 had a moderate educational level.

Aligning with Baseline Class 3, Follow-up Class 3 was the largest (90.5% of sample), showing a low probability of CHR-P symptoms (Fig. 2b), along with the lowest rates of psychosocial deficits and axis-I disorders among Follow-up Classes. Moreover, Follow-up Class 3 had the highest rate of regular employment, the lowest divorce rate and, newly, the highest educational level (Table 3).

Finally, similarly to Baseline Classes, the Follow-up Classes did not differ in distribution of sex, nationality, or family history of mental disorders, and, additionally, also not in age.

### Movement between classes from baseline to follow-up

**Figure 3.**
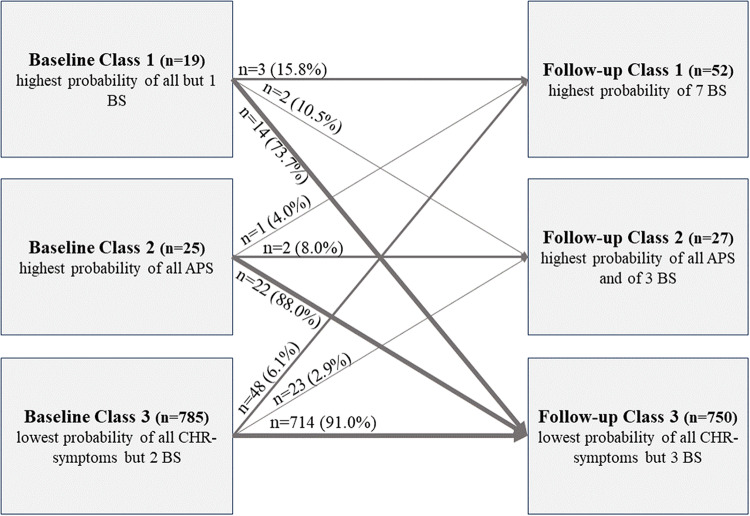
Changes of class membership over time.

In absolute terms, more participants (*n* = 79) were included in the two more impaired Classes 1 and 2 at follow-up than had been at baseline (*n* = 44). However, less than a quarter (*n* = 8) of Baseline Class 1 or 2 members stayed in, or moved to, the corresponding Follow-up Classes 1 or 2. Instead, the majority of participants in the more impaired Baseline Classes 1 and 2 (73.7% and 88.0%, respectively) moved to the ‘healthy’ Follow-up Class 3, which still included most (91.0%) members of the ‘healthy’ Baseline Class 3 (Fig. 3). In contrast, 9.0% of members (*n* = 71) of the least impaired Baseline Class 3 moved to the more impaired Follow-up Classes 1 or 2 (6.1% and 2.9%, respectively; Fig. 3).

### Demographic and socio-economic characteristics across CHR-P classes

The classes differed little in distribution of sex, nationality, family history of psychiatric disorders and age, although participants in Baseline Class 2 were the oldest at both baseline and follow-up. Across time points, Class 3 had the highest rate of regular employment.

The distribution of education and marital status showed more variation. While there was less distinction between Baseline Classes at either time point, Follow-up Class 3 showed significantly higher education and lower divorce rates than Follow-up Classes 2 and 1. In turn, Follow-up Class 2 participants were most frequently unmarried, while Follow-up Class 1 members were most often separated. Finally, the education level in Follow-up Class 2 was slightly higher than in Follow-up Class 1.

### Changes and class characteristics of CHR-P symptoms

CHR-P symptom profiles showed some relevant changes across classes and time points.

At follow-up, Baseline Classes 1 and 3 showed a more than twofold increase in the rate of (attenuated) hallucinations. As a result, Baseline Class 1, whose members also exhibited increased rates of thought blockage and derealization, now showed a similar rate of (attenuated) hallucinations to Baseline Class 2. Despite this, the rate of (attenuated) hallucinations in Baseline Class 3 remained significantly smaller than in both symptomatic classes. Similarly, perceptual BS had more than doubled, with the increase being particularly pronounced in Baseline Class 3, thus leading to a lack of significant class differences in perceptual BS at follow-up. In summary, while perceptual symptoms had little influence on class identification at baseline, their increase at follow-up turned them into highly influential symptoms for the definition of both symptomatic classes.

Conversely, unusual thought content (SIPS P1), which had been highly influential on class separation at baseline, did not maintain this role for Class 1 at follow-up. However, it remained highly influential for Class 2, which continued to show the overall highest rate of any APS/BIPS at this time point. Newly, Follow-up Class 2 also showed the highest prevalence rates of four BS: inability to divide attention, disturbance of receptive speech, decreased ability to discriminate between ideas/perception and fantasy/true memories, and derealization. Additionally, visual perception disturbances occurred more frequently in Follow-up Class 2, although still less frequently than in Follow-up Class 1. This was a notable change compared to the baseline assessment, where no BS had been most frequent in Class 2, although disturbance of receptive speech, derealization and thought pressure had occurred frequently.

## Discussion

To the best of our knowledge, this is the first longitudinal study of classes of a comprehensive collection of CHR-P symptoms in the community, and the first that not only examines homogeneous classes of individuals at baseline but also their stability and change in class membership over time.

### Symptomatic characteristics of classes over time

Our three-class solution aligns with earlier LCA studies of UHR patients, which predominantly reported three classes (Healey *et al.*, 2018; Ryan *et al.*, 2018), though some found four (Valmaggia *et al.*, 2013) or five (Ryan *et al.*, 2018). Most focused solely on UHR patients, with only one (Healey *et al.*, 2018) including healthy controls, making it most comparable to ours. Healey et al. also identified a three-class solution with a ‘mild’ class similar to our Class 3. However, unlike our study, APS/BIPS were not highly influential in their results, possibly due to UHR criteria favouring positive symptoms. The influential role of negative symptoms in earlier studies contrasts with our study’s emphasis on APS/BIPS, possibly due to the exclusion of negative symptoms in our analysis.

The differentiation of symptomatic classes in our community study into one characterized mainly by APS/BIPS, and one characterized mainly by BS, is in line with previous reports of SIPS positive items and BS mostly clustering in different classes (Jimeno *et al.*, 2020, 2022). In a recent network cluster analysis (Jimeno *et al.*, 2020), only hallucinatory symptoms (SIPS-P4) had joined the cluster of BS; this being broadly in line with Follow-up Class 1 that was characterized by seven BS and (attenuated) hallucinations (SIPS-P4). However, APS/BIPS and BS were best separated at baseline.

Baseline class characteristics remained consistent over time, with notable exceptions, particularly an increase in perceptual symptoms in Class 1 at follow-up. Given earlier findings linking (attenuated) hallucinations and BS to younger age (Schimmelmann *et al.*, 2015; Schultze-Lutter *et al.*, 2017, 2020a, 2020b; Schultze-Lutter and Schmidt, 2016; Walger *et al.*, 2020), this increase was unexpected. Future studies should examine features related to this increase to better understand the course of perceptual symptoms in the community. Further, the cross-class occurrence of thought pressure, derealization and visual perception disturbances, as well as suspiciousness/persecutory ideas, may be attributed to their transdiagnostic nature, not observed in other CHR-P symptoms or criteria (Schultze-Lutter *et al.*, 2022).

### Associated features over time and class solutions

Interestingly, despite the sample’s generally reduced symptom load at follow-up, which is in line with other studies (Bergé *et al.*, 2024; Salazar de Pablo *et al.*, 2022), the number of members in the two symptomatic classes increased from baseline to follow-up. Comparing the socio-demographic and clinical features between assessment times and LCA solutions, however, revealed some small changes in distribution of axis-I disorders that tended to be most frequent in Class 1, and least frequent in Class 3 over time, and across solutions. This was despite a decline in axis-I disorders over time, in particular in affective and other disorders (i.e., eating disorders, somatoform disorders, etc.), that aligns with reports of a decline of comorbid mental disorders over time from clinical CHR-P samples (Solmi *et al.*, 2023). The combination of CHR-P symptoms and non-psychotic mental disorder is considered a particularly ‘risky’ form of CHR-P state, with poorer outcome compared to CHR-P symptoms in isolation (Hasmi *et al.*, 2021). This might explain the poor outcome of Baseline Class 1 members who had the highest rate of baseline axis-I disorder and, at follow-up, had the highest rates of axis-I disorders and functional deficits.

Functional deficits demonstrated little change in overall frequency over time, and were generally lowest in Class 3, but differed between the symptomatic classes in distribution over time and solutions. While functional deficits were similarly frequent in Baseline Classes 1 and 2 at baseline, at follow-up, they were most frequent in Baseline Class 1 and in Follow-up Class 2. This lack of a significant improvement in functioning is in contrast to reports from follow-up studies of CHR-P samples that commonly report significant functional improvement over time (Salazar de Pablo *et al.*, 2022). The difference in findings may be related to a difference in samples, with far fewer participants with functional deficits in our community sample and/or to the assessment of functioning – dichotomized data in our study, and continuous raw data in most clinical studies (Salazar de Pablo *et al.*, 2022). Overall, the generally maintained disadvantages of the symptomatic classes over time, despite symptomatic improvements, underscore the importance of preventive approaches not only with regard to mental disorders but also functional deficits and vocational-educational disadvantages (Campion *et al.*, 2012; Porru *et al.*, 2023).

### Membership changes between Baseline and Follow-up Classes

In line with the general symptomatic, clinical and socio-demographic stability of Class 3 over time, this class showed the lowest rate of changes into any symptomatic class, indicating that most participants remained ‘healthy’ over time. Furthermore, the highest rate of class membership changes of the two symptomatic classes were into Follow-up Class 3, indicating health improvement and an attenuation of most CHR-P symptoms over time (Addington *et al.*, 2020). In absolute numbers, however, more participants moved from the large Baseline Class 3 into one of much smaller symptomatic Follow-up Classes; with a third of them into Follow-up Class 1. The unexpected transition from ‘healthy’ to psychopathological symptoms suggests that even those with few or no symptoms may be at risk for later development of CHR-P symptoms. This broadens the focus of early detection efforts beyond solely ‘at-risk’ individuals with CHR-P symptoms, prompting exploration of hidden factors including (neuro)biological and psychosocial influences, such as inflammatory processes and negative life events (de Koning *et al.*, 2022; Trotta *et al.*, 2015). Understanding these factors beyond genetic predisposition is crucial for comprehensively addressing psychopathology development.

Follow-up Class 1 also showed higher membership stability compared to Follow-up Class 2 (16% vs. 8%). This broadly aligns with reported changes of CHR-P criteria in a clinical sample of an early detection service over 1–10-year follow-up (Michel *et al.*, 2018a), in which most non-converters had remitted from CHR-P status (72%), and more non-converters with the baseline BS criterium ‘Cognitive Disturbances’ than with baseline UHR criteria maintained their risk status (18% vs. 12%). Furthermore, 91% of CHR-P-negative patients remained CHR-P-negative (Michel *et al.*, 2018a). Overall, our results support the fluctuating nature of CHR-P symptoms.

### Practical recommendations

Based on our findings, we propose several practical steps to improve early detection and intervention for CHR-P symptoms. Community-based prevention efforts should prioritize targeted mental health literacy programmes aimed at the public, healthcare providers and educators. These programmes should focus on increasing awareness of early CHR-P symptoms – such as perceptual disturbances, cognitive difficulties and social withdrawal – while addressing stigma to promote timely help-seeking.

In primary care settings, integrating brief and validated CHR-P screening tools, such as the Prodromal Questionnaire-Brief (PQ-B; Loewy *et al.*, 2011) or the Community Assessment of Psychic Experience (CAPE; Mossaheb *et al.*, 2012), into routine clinical practice can facilitate earlier identification of individuals at risk. Training primary care professionals to recognize key indicators of CHR-P, including comorbid mood or anxiety symptoms, is essential for appropriate referral to specialized services.

For individuals with functional impairments or symptomatic profiles, targeted interventions such as cognitive-behavioural therapy, stress management techniques and resilience-building programmes should be offered. Family psycho-education and support can also play a critical role in improving social and functional outcomes.

Given the heterogeneity of CHR-P presentations, personalized preventive strategies are crucial. These should be informed by comprehensive assessments of psychosocial factors (e.g., trauma history, family dynamics), neurocognitive deficits (e.g., executive dysfunction) and biological risk markers (e.g., sleep disturbances or neuroinflammation). Tailoring interventions to individual risk profiles increases their precision and effectiveness.

Lastly, longitudinal monitoring of individuals with mild or subthreshold symptoms is vital to detect emerging risk states. This can be achieved through structured follow-ups and the use of digital tools, such as Ecological Momentary Assessment and telehealth platforms, which allow real-time tracking of symptom trajectories and functional outcomes. Such continuous monitoring enables adaptive and timely interventions that may prevent progression to fully manifest psychosis.

By implementing these strategies, we can enhance the early identification of CHR-P states, provide timely and individualized interventions, and ultimately improve long-term outcomes for at-risk individuals.

### Strengths and limitations

Our symptom selection is both a strength and limitation. While our study is the first LCA study to include the full spectrum of CHR-P symptoms (Schultze-Lutter *et al.*, 2015), it did not include non-CHR-P-relevant symptoms, such as negative symptoms, which have been shown to differentiate classes (Healey *et al.*, 2018; Ryan *et al.*, 2018; Valmaggia *et al.*, 2013). However, the shift towards a stepwise psychosis detection approach, assessing CHR-P criteria first (Schultze-Lutter and Meisenzahl, 2023, 2024; Woods *et al.*, 2023), suggests our classes may reflect early diagnostic steps. Strengths of our study include clinical assessments by trained psychologists and a large, well-representative sample (Schultze-Lutter *et al.*, 2018). Still, the small size of symptomatic classes warrants caution in interpretation. Additionally, like earlier studies, we did not account for the impact of treatment, which could have influenced class development. Treatment may be particularly influential, as higher symptom loads often lead to increased help-seeking (Michel *et al.*, 2019).

### Conclusion and future directions

Our results suggest that CHR-P symptoms cluster similarly in the community as in clinical samples, despite their fluctuation over time, underpinning the largely distinct and, therefore, complementary nature of the BS and symptomatic UHR approaches (Schultze-Lutter *et al.*, 2020a). In addition, the association of the two symptom classes with axis-I disorders and functional deficits emphasizes the clinical significance of CHR-P symptoms beyond a potential bias towards higher clinical relevance in patient samples (Fusar-Poli *et al.*, 2016; Ruhrmann *et al.*, 2010; Schmidt *et al.*, 2015).

These results emphasize the importance of preventive measures in general, and point to the need to improve mental health literacy in relation to CHR-P states and symptoms in the community (Kelly *et al.*, 2007). All the more so, as compared to other mental disorders, such as depression (Svensson and Hansson, 2016), there is a significant lack of knowledge, misunderstanding and negative stereotyping of psychotic disorders, including their symptoms and risk stages (Doll *et al.*, 2022; Goodwin, 2014; O’Keeffe *et al.*, 2016; Patel, 2004), in the healthcare system, the public and the media. Even those affected often lack a clear understanding of the CHR-P condition, which delays their help-seeking (Haidl *et al.*, 2019). At the clinical level, improved stepwise diagnostic approaches drawing from broad psychopathological, resilience, neurocognitive and biogenetic assessments for improved risk profiling for various outcomes and risk-adapted treatments should enable a more personalized, broader prevention approach that better fits the need of different person classes (Schultze-Lutter and Meisenzahl, 2023, 2024; Worthington and Cannon, 2021).

## Supporting information

Michel et al. supplementary material Michel et al. supplementary material

## Data Availability

Data will not be directly available on a public repository or in the supplements. However, it can be made available on request via the corresponding author (C.M.).

## Supplementary material

The supplementary material for this article can be found at https://doi.org/10.1017/S2045796024000891.
