# Supplementary material for: Three-year course of clinical high-risk symptoms for psychosis in the community: a latent class analysis
Source: Epidemiol Psychiatr Sci. 2025 Jan 13;34:e3. doi: 10.1017/S2045796024000891 (PMC11735126; doi:10.1017/S2045796024000891)
Supplement: Michel et al. supplementary material [file S2045796024000891sup001.docx]

# Supplementary Material to:

**Course of Clinical High-Risk Symptoms for Psychosis in the Community: A Latent Class Analysis with a focus on movement between subgroups over time**

## Chantal Michel, Naweed Osman, Giulia Rinaldi, Benno G. Schimmelmann, Jochen Kindler, and Frauke Schultze-Lutter

**Contents:**

**eText 1:** The Bern Epidemiological At-Risk (BEAR) study - details on study design

**eText 2:** Details on recruitment of sample

**eTable 1:** Clinical high-risk for psychosis (CHR-P) symptoms and criteria

**eTable 2:** Comparison of sociodemographic and clinical characteristics at baseline and follow-up (N=829)

**eTable 3:** Evaluating of the class solutions at baseline

**eTable 4:** Evaluation of the class solutions at follow-up

**eText 1: The Bern Epidemiological At-Risk (BEAR) study – details on study design**

At baseline, a representative sample of the Bernese general population was obtained using a stratified sampling method. Participants were randomly selected from the approximately 310,000 predominantly Caucasian 16 to 40 years old residents of the semi-rural Canton Bern.

The BEAR-sample was evaluated during a semi-structured telephone interview. Excellent concordance rates (78-100%) were found for telephone and face-to-face assessment for the used clinical interviews in a feasibility study that was carried out prior to the BEAR-study baseline assessment.^1^

Eligibility criteria were inclusion in the selected age range, main residency in Canton Bern (i.e. having a valid address in the Canton and not being abroad during the assessment period), and an available telephone number.

First telephone contact was attempted two weeks after sending eligible participants a one-page information letter, meant to increase response rates, and explaining the study goals and procedure, as well as the incentives for participation.

Participation in the telephone interview after receiving exhaustive information about the study was considered as giving informed consent. Eligible participants that could not be reached after up to 100 calls over several months, at different times and days including Saturdays, were considered as unknown eligible.

Further exclusion criteria were (i) a lifetime diagnosis of psychosis^2^ and (ii) insufficient fluency in German, French or English. If respondents met one of these criteria, their interview was interrupted prematurely. On average, the semi-structured interviews lasted 43 minutes (SD: 20 minutes; range: 20–225 minutes).

To ensure an excellent assessment quality, clinical psychologists conducted the telephone interviews after three months of intensive training, and were provided with weekly supervision by F. Schultze-Lutter and C. Michel.^2^

**eText 2: BEAR-study - details on recruitment of sample and representativeness**

*Baseline*

At baseline, participants were first recruited from 06/2011 to 11/2014. Completed interviews were 2,683, with a contact rate of 94.8% and a response rate of 63.4%. Compared to the 16- to 40-year-old general population of Bern, the eligible sample was negligibly older, but this difference was mainly based on a higher non-significant number of available telephone numbers (landlines) in 36- to 40-year-olds.

For the 2,683 participants who completed the interview, negligible differences were detected in age distribution, but not gender, nationality or marital status, when compared to the 16- to 40-year-old general population of Bern. They were therefore considered to be a representative sample of their age group.^3^

*Follow-up*

The BEAR study demonstrated a point-prevalence of CHR symptoms of 13.8%. Of the 2,857 participants, 23.1% (n=659) reported at least one CHR symptom irrespective of onset or frequency requirements of CHR criteria in the baseline assessment; 97.9% of these (n=645) agreed to be re-contacted for a follow-up. This sub-sample formed the main target group (RISK) for the follow-up assessment. To this, a control group (CONTROL) of 645 persons not having reported any CHR symptom at baseline was selected that was matched to RISK participants for (i) sex (ii) age, and (iii) education at baseline. In case of refusal or failure to renew contact, a CONTROL subject was replaced by another match to the respective RISK subject.

From 06/2015 to the conclusion of the study in 03/2018, a total of 1,028 participants was re-contacted (incl. replacement of CONTROL subjects) for the follow-up assessment (median follow-up 39 months). The recruitment rates for the FU according to the American Association for Public Opinion Research with regard to all n=834 interviewees were as follows:

- the contact rate was 74.8%,
- the cooperation rate was 87.8%,
- the refusal rate was only 9.1%, and
- the response rate was 65.6% (829 non-conversions, 5 conversions^4^ to psychosis, 5 partial interviews).

The sample used for the analyses consisted of the N=829 non-converters.

**eTable 1: Clinical high-risk for psychosis (CHR-P) symptoms**

| **Ultra-high risk (UHR) criteria** according to the SIPS |
| --- |
| A. ‘Brief Intermittent Psychotic Symptoms’ (BIPS)  ⮊ At least any 1 of the following SIPS P-items scored 6 ‘severe and psychotic’   - P1 Unusual Thought Content / Delusional Ideas - P2 Suspiciousness / Persecutory Ideas - P3 Grandiose Ideas - P4 Perceptual Abnormalities / Hallucinations - P5 Disorganized Communication   ⮊ First appearance in the past three months  ⮊ Present for at least several minutes per day at a frequency of at least once per month but less than 7 days |
| B. ‘Attenuated Positive Symptoms’ (APS)  ⮊ At least any 1 of the following SIPS P-items scored 3 ‘moderate’ to 5 ‘severe but not psychotic’   - P1 Unusual Thought Content / Delusional Ideas - P2 Suspiciousness / Persecutory Ideas - P3 Grandiose Ideas - P4 Perceptual Abnormalities / Hallucinations - P5 Disorganized Communication   ⮊ First appearance within the past year or current rating one or more scale points higher compared to 12 months ago  ⮊ Symptoms have occurred at an average frequency of at least once per week in the past month |
| C. ‘Genetic Risk and Deterioration’ Syndrome  (1) Patient meets criteria for Schizotypal Personality Disorder according to SIPS  (2) Patient has 1^st^ degree relative with a psychotic disorder  (3) Patient has experienced >30% drop in global assessment of functioning (GAF) score over the last month compared to 12 months ago  ⮊ [1 and 3] or [2 and 3] or all are met. |
| **Basic symptom (BS) criteria** |
| Risk criterion ‘Cognitive-Perceptive Basic Symptoms’ (COPER)  ⮊ At least any 1 of the following BS with a SPI-A score of ≥3 within the last 3 months:   - Thought interference (BS6; SPI-A C2) - Thought pressure (BS7; SPI-A D3) - Disturbance of receptive speech (BS5; SPI-A C4) - Thought perseveration (BS8; SPI-A O1) - Thought blockages (BS9; SPI-A C3) - Decreased ability to discriminate between ideas / perception and fantasy / true memories (BS10; SPI-A O2) - Unstable ideas of reference (BS11; SPI-A D4) - Derealisation (BS12; SPI-A O8) - Visual perception disturbances (excluding hypersensitivity to light or blurred vision) (BS13; SPI-A O4, F3, D5) - Acoustic perception disturbances (excluding hypersensitivity to sounds) (BS14=SPI-A O5, F5)   ⮊ First occurrence ≥12 months ago |
| High-risk criterion ‘Cognitive Disturbances’ (COGDIS)  ⮊ At least any 2 of the following BS with a SPI-A score of ≥3 within the last 3 months:   - Inability to divide attention (BS1; SPI-A B1) - Captivation of attention by details of the visual field (BS2; SPI-A O7) - Disturbances of abstract thinking (BS3; SPI-A O3) - Disturbance of expressive speech (BS4; SPI-A C5) - Disturbance of receptive speech (BS5; SPI-A C4) - Thought interference (BS6; SPI-A C2) - Thought pressure (BS7; SPI-A D3) - Thought blockages (BS9; SPI-A C3) - Unstable ideas of reference (BS11; SPI-A D4) |

| eTable 2. Comparison of sociodemographic and clinical characteristics at baseline and follow-up (N=829) | | | | | | |
| --- | --- | --- | --- | --- | --- | --- |
|  | | Baseline | | Follow-up | | Statistics |
|  | | n | % [significant standardized residuals] | n | % [significant standardized residuals] |  |
| Age (mean±SD, median, range) | | 29.83±7.68, 32, 16-41 | | 33.25±7.77, 35, 19-45 | | F=81.39, df=1, p<0.001, η^2^=0.047 |
| Sex (male) | | 441 | 53.2 | 441 | 53.2 | χ^2^=0, df=1, p=1, Cramer's V=0 |
| Nationality (Swiss) | | 798 | 96.3 | 805 | 97.1 | χ^2^=0.677, df=1, p=0.411, Cramer's V=0.024 |
| Highest education | | | | | | |
|  | ISCED level 0-2 | 15 | 1.8 | 15 | 1.8 | χ^2^=0, df=4, p=1, Cramer’s V=0 |
|  | ISCED level 3 | 117 | 14.1 | 117 | 14.1 |  |
|  | ISCED level 4-5 | 392 | 47.3 | 392 | 47.3 |  |
|  | ISCED level 7 | 297 | 35.8 | 297 | 35.8 |  |
|  | ISCED level 8 | 8 | 1.0 | 8 | 1.0 |  |
| Current employment | | | | | | |
|  | unemployed | 21 | 2.5 | 19 | 2.3 | χ^2^=3.772, df=4, p=0.496, Cramer’s V=0.048 |
|  | sheltered employment | 1 | 0.1 | 4 | 0.5 |  |
|  | temporary employment | 8 | 1.0 | 7 | 0.8 |  |
|  | regular full- and part-time employment | 798 | 96.3 | 795 | 95.9 |  |
|  | other | 1 | 0.1 | 4 | 0.5 |  |
| Marital status | | | | | | |
|  | single | 470 | 56.7 [2.27] | 424 | 51.1 [-2.27] | χ^2^=7.402, df=5, p=0.160, Cramer’s V=0.067 |
|  | married/civil union | 335 | 40.4 | 368 | 44.4 |  |
|  | separated | 11 | 1.3 | 14 | 1.7 |  |
|  | divorced | 10 | 1.2 | 19 | 2.3 |  |
|  | widowed | 1 | 0.1 | 2 | 0.2 |  |
|  | other | 2 | 0.2 | 2 | 0.2 |  |
| Family history of psychiatric disorders | | 352 | 42.6 [-2.81] | 409 | 49.5 [2.81] | χ^2^=7.641, df=1, p=0.006, Cramer's V=0.069 |
| SOFAS deficit (SOFAS<70) | | 62 | 7.5 | 58 | 7.0 | χ^2^=0.081, df=1, p=0.776, Cramer's V=0.009 |
| Any current axis-I disorder^a^ | | 141 | 17.0 [2.12] | 110 | 13.3 [-2.12] | χ^2^=4.225, df=1, p=0.040, Cramer's V=0.052 |
|  | Any affective disorder | 54 | 6.5 [4.04] | 20 | 2.4 [-4.04] | χ^2^=15.404, df=1, p<0.001, Cramer's V=0.099 |
|  | Any anxiety disorder | 97 | 11.7 | 98 | 11.8 | χ^2^=0, df=1, p=1, Cramer's V=0.002 |
|  | Any other disorder | 36 | 4.3 [3.34] | 13 | 1.6 [-3.34] | χ^2^=10.178, df=1, p=0.001, Cramer's V=0.082 |
| CHR-P symptoms | | | | | | |
|  | P1: Unusual thought content/delusional ideas | 67 | 8.1 [2.26] | 44 | 5.3 [-2.26] | χ^2^=4.673, df=1, p=0.030, Cramer's V=0.056 |
|  | P2: Suspiciousness/persecutory ideas | 29 | 3.5 [3.93] | 6 | 0.7 [-3.93] | χ^2^=14.127, df=1, p<0.001, Cramer's V=0.096 |
|  | P3: Grandiose ideas | 4 | 0.5 | 2 | 0.2 | χ^2^=0.167, df=1, p=0.687, Cramer's V=0.020 |
|  | P4: Perceptual abnormalities/ hallucinations | 42 | 5.1 [-4.05] | 86 | 10.4 [4.05] | χ^2^=15.654, df=1, p<0.001, Cramer's V=0.099 |
|  | P5: Disorganized communication | 15 | 1.8 [2.25] | 5 | 0.6 [-2.25] | χ^2^=4.099, df=1, p=0.040, Cramer's V=0.055 |
|  | BS1: Inability to divide attention (SPI-A B1) | 6 | 0.7 | 4 | 0.5 | χ^2^=0.101, df=1, p=0.753, Cramer's V=0.016 |
|  | BS2: Captivation of attention by details of the visual field (SPI-A O7) | 11 | 1.3 [2.51] | 2 | 0.2 [-2.51] | χ^2^=4.962, df=1, p=0.022, Cramer's V=0.062 |
|  | BS3: Disturbances of abstract thinking (SPI-A O3) | 3 | 0.4 | 0 | 0.0 | χ^2^=1.336, df=1, p=0.250, Cramer's V=0.043 |
|  | BS4: Disturbances of expressive speech (SPI-A C5) | 32 | 3.9 [3.64] | 9 | 1.1 [-3.64] | χ^2^=12.104, df=1, p<0.001, Cramer's V=0.089 |
|  | BS5: Disturbances of receptive speech (SPI-A C4) | 1 | 0.1 | 4 | 0.5 | χ^2^=0.802, df=1, p=0.374, Cramer's V=0.033 |
|  | BS6: Thought interference (SPI-A C2) | 8 | 1.0 | 9 | 1.1 | χ^2^=0, df=1, p=1, Cramer's V=0.006 |
|  | BS7: Thought pressure (SPI-A D3) | 18 | 2.2 | 9 | 1.1 | χ^2^=2.41, df=1, p=0.119, Cramer's V=0.043 |
|  | BS8: Thought perseveration (SPI-A O1) | 2 | 0.2 | 1 | 0.1 | χ^2^=0, df=1, p=1, Cramer's V=0.014 |
|  | BS9: Thought blockages (SPI-A C3) | 49 | 5.9 [2.99] | 24 | 2.9 [-2.99] | χ^2^=8.254, df=1, p=0.004, Cramer's V=0.074 |
|  | BS10: Decreased ability to discriminate between ideas & perception, fantasy & true memories (SPI-A O2) | 9 | 1.1 [2.12] | 2 | 0.2 [-2.12] | χ^2^=3.295, df=1, p=0.065, Cramer's V=0.052 |
|  | BS11: Unstable ideas of reference (SPI-A D4) | 25 | 3.0 [2.77] | 9 | 1.1 [-2.77] | χ^2^=6.756, df=1, p=0.008, Cramer's V=0.068 |
|  | BS12: Derealisation (SPI-A O8) | 20 | 2.4 [2.06] | 9 | 1.1 [-2.06] | χ^2^=3.51, df=1, p=0.059, Cramer's V=0.051 |
|  | BS13: Visual perception disturbances (SPI-A O4, F3, D5) | 28 | 3.4 | 43 | 5.2 | χ^2^=2.884, df=1, p=0.089, Cramer's V=0.045 |
|  | BS14: Acoustic perception disturbances (SPI-A O5, F5) | 29 | 3.5 [-4.98] | 79 | 9.5 [4.98] | χ^2^=23.781, df=1, p<0.001, Cramer's V=0.122 |

*Note:* SOFAS: Social and Occupational Functioning Assessment Scale.

In **[bold]**, cells with standardized residuals ≥|1.96|. This equals significant deviation from the expected cell frequency. 1.96 indicates that the number of cases in that cell is significantly larger than would be expected if the null hypothesis were true, with a significance level of 0.05. An adjusted residual that is less than -1.96 indicates that the number of cases in that cell is significantly smaller than would be expected if the null hypothesis were true.

**eTable 3: Evaluation of the class solutions at baseline**

|  | *Model fit criteria* | | *Diagnostic criteria* |
| --- | --- | --- | --- |
| Model | AIC | BIC | Relative entropy |
| 2 class | 3346.689 | **3530.777** | 0.885 |
| 3 class | **3332.746** | 3611.239 | 0.911 |
| 4 class | 3338.659 | 3711.556 | **0.970** |

*Note: N*=829. AIC: Akaike information criterion; BIC: Bayesian information criterion. Items in bold represent the best model fit.

**eTable 4: Evaluation of the class solutions at follow-up**

|  | *Model fit criteria* | | *Diagnostic criteria* |
| --- | --- | --- | --- |
| Model | AIC | BIC | Relative entropy |
| 2 class | 3346.689 | 3530.777 | 0.884 |
| 3 class | **2730.968** | **2995.301** | 0.779 |
| 4 class | 3338.506 | 3711.403 | **0.999** |

*Note: N*=829. AIC: Akaike information criterion; BIC: Bayesian information criterion. Items in bold represent the best model fit.

**References Supplementary Material**

1. Michel C, Schimmelmann BKE, Kupferschmid S, Siegwart M, Schultze-Lutter F. Reliability of telephone assessments of at-risk criteria of psychosis: a comparison to face-to-face interviews. *Schizophr Res*. 2014;153(1-3):251-253.

2. Michel C, Schimmelmann BG, Schultze‐Lutter F. Demographic and clinical characteristics of diagnosed and non‐diagnosed psychotic disorders in the community. *Early Interv Psychiatry*. 2018;12(1):87-90.

3. Schultze-Lutter F, Michel C, Ruhrmann S, Schimmelmann BG. Prevalence and clinical relevance of interview-assessed psychosis-risk symptoms in the young adult community. *Psychol Med*. 2018;48(7):1167-1178. doi:10.1017/S0033291717002586

4. Schultze-Lutter F, Schimmelmann BG, Michel C. Clinical high-risk of and conversion to psychosis in the community: A 3-year follow-up of a cohort study. *Schizophr Res*. 2021;228:616-618. doi:10.1016/j.schres.2020.11.032
